# Divergent dynamics in whole-body regeneration and larval development of sponge *Haliclonasimulans*: cytobiology, microbiome, and transcriptomics

**DOI:** 10.1016/j.isci.2026.115344

**Published:** 2026-03-13

**Authors:** Chenzheng Jia, Beibei Zhang, Bifu Gan, Yuqing Zhao, Xin Lin, Jun Chen, Jing Zhao

**Affiliations:** 1 College of Ocean and Earth Sciences, Xiamen University, Xiamen 361005, China; 2 Fujian Ocean Innovation Center, Xiamen 361102, China; 3 Fujian Provincial Key Lab of Coastal Basin Environment, Fujian Polytechnic Normal University, Fuqing 350300, China

**Keywords:** developmental biology, omics

## Abstract

Sponges are capable of rebuilding functional individuals from cell aggregates (primmorphs), a process termed whole-body regeneration that morphologically parallels larval development. To systematically compare these processes, we established an *in vitro* primmorph regeneration model in *Haliclona simulans* and performed multi-level analyses across planktonic, settled, and metamorphic stages. Although both processes formed similar structures (e.g., the aquiferous system), planktonic primmorphs exhibited a reduced stem cell proportion (archeocyte/choanocyte), along with the increase of seemingly dedifferentiating cells and vacuolar cells. Microbiome diverged: while sharing core symbionts (e.g., *AqS1*), primmorphs enriched *Tenacibaculum* and *Vibrio* species during remodeling process. Transcriptomics revealed distinct signatures: regeneration upregulated genes potentially related to DNA repair and dedifferentiation but downregulated stem cell markers. Our integrative study indicates that regeneration and development constitute distinct processes, achieving similar functional outcomes via divergent cellular, microbial, and molecular profiles that provides a foundational framework for future mechanistic studies of regeneration.

## Highlights

• Sponge regeneration and larval development share morphological outcomes
• Regeneration has fewer stem cells, more vacuolar cells, and possible dedifferentiation
• Both share core symbionts *AqS1*, but regeneration enriches *Vibrio* and *Tenacibaculum*
• Regeneration downregulates stem cell markers but upregulates DNA repair-related genes

## Graphical abstract

**Figure:**
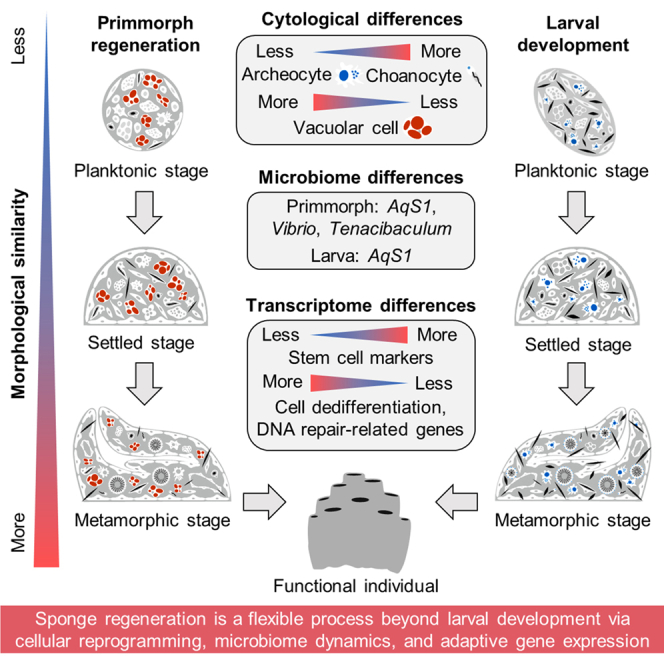

## Introduction

Regeneration, the restoration of damaged or missing body parts, is widespread but highly variable among organisms.^1^^,^^2^ Although regeneration and development represent different physiological phenomena occurring at different stages of life, in certain organisms like salamanders and zebrafish, the newly generated organs during regeneration exhibit morphological and functional resemblance to the original organs formed during development.^3^^,^^4^ While this similarity in outcomes makes it easy to speculate that similar cellular and molecular mechanisms may be involved in both regenerative and developmental processes, the extent of this mechanistic overlap remains debatable.^5^^,^^6^ For over a century, researchers have been comparing regeneration with embryonic development in hopes of understanding and unlocking the ability to regenerate tissues or organs from a developmental perspective.^4^^,^^7^^,^^8^^,^^9^^,^^10^ Recent studies on porifera,^7^ sea anemone,^8^ brittle star,^9^ crustaceans,^10^ and zebrafish^3^ have highlighted similarities as well as differences between regeneration and development. However, it still remains unclear to what extent regeneration recapitulates the biological processes of development. Currently, there are several challenges in the field of regenerative biology, (1) many studies have focused solely on comparing the expression levels of individual genes during regeneration versus development, without considering overall comparisons,^11^^,^^12^ (2) most research has primarily concentrated on the repair of trauma rather than whole-body regeneration,^3^^,^^7^^,^^8^^,^^9^ (3) our current understanding of regenerative mechanisms is derived from only a limited number of model organisms, which restricts our comprehensive knowledge about this process.^13^

Sponges offer valuable biological material for studying the intricate relationship between regeneration and development for several reasons. Firstly, sponges represent one of the most primitive multicellular organisms and are considered the most ancient metazoans.^14^^,^^15^ Elucidating their regenerative mechanisms could provide insight into the evolutionary origins of regeneration in multicellular animals. Secondly, due to their sessile bottom-dwelling nature, sponges inhabit diverse aquatic habitats worldwide and play an important role in marine benthic ecosystems.^16^^,^^17^ To adapt to frequent environmental disturbances such as predation, storms, bottom-fishing, etc., sponges evolve remarkable regenerative capabilities ranging from the repair of trauma to whole-body regeneration.^18^^,^^19^ Unlike other model organisms, even small tissue fragments or cell aggregates known as “primmorphs” can initiate the process of whole-body regeneration in sponges within a short period of time,^7^^,^^20^^,^^21^^,^^22^^,^^23^ probably representing an ancestral trait lost in mammals. Thirdly, adult sponges, especially *H. simulans*, which we are interested in, release a large number of larvae during their reproductive stage. These larvae rapidly develop into juvenile sponges within a week through settlement and metamorphosis.^24^ Numerous studies have extensively documented the morphology of larval development.^25^ Fourthly, coevolution spanning over 890 million years leads to the formation of sponge holobionts-synergistic communities where symbiotic microorganisms account for 40%–60% of the sponge’s biomass and play a crucial role in its survival and health.^26^ Investigating sponge regeneration and development from a microbial perspective provides valuable insights into understanding microbe-host interactions. These characteristics make sponges an exceptional model for the comparative analysis of regeneration and development.

This study employs primmorph reaggregation—a radical form of whole-body regeneration from dissociated adult cells—as a model to systematically compare with larval development in the sponge *H. simulans*, a dominant sponge species ubiquitous along the southeastern coast of China. We integrated morphological, microbiome, and transcriptome profiling to analyze both processes. Initially, we established a sponge primmorph culture system capable of reconstructing complete functional individuals and defined several key regeneration stages based on morphological criteria. Subsequently, we collected samples from three corresponding stages (planktonic, settled, and metamorphic stages) during both regeneration and larval development for high-throughput sequencing to compare the microbiome and transcriptome underlying their seemingly morphogenetic process. Our results reveal that although primmorph regeneration and larval development share morphological similarities, conserved symbionts, and partially overlapping gene expression, they exhibit fundamental differences in cellular reconstruction dynamics (e.g., stem cell availability and possible cell dedifferentiation), microbial community transitions, and gene expression patterns (e.g., differential regulation of DNA repair activation). Elucidating these divergences not only provides a foundational framework and testable hypotheses for future mechanistic studies of regeneration but also offers insights into the evolution of regenerative capacity in metazoans.

## Results

### Morphological and cytological comparison between primmorph regeneration and larval development

**Figure 1:**
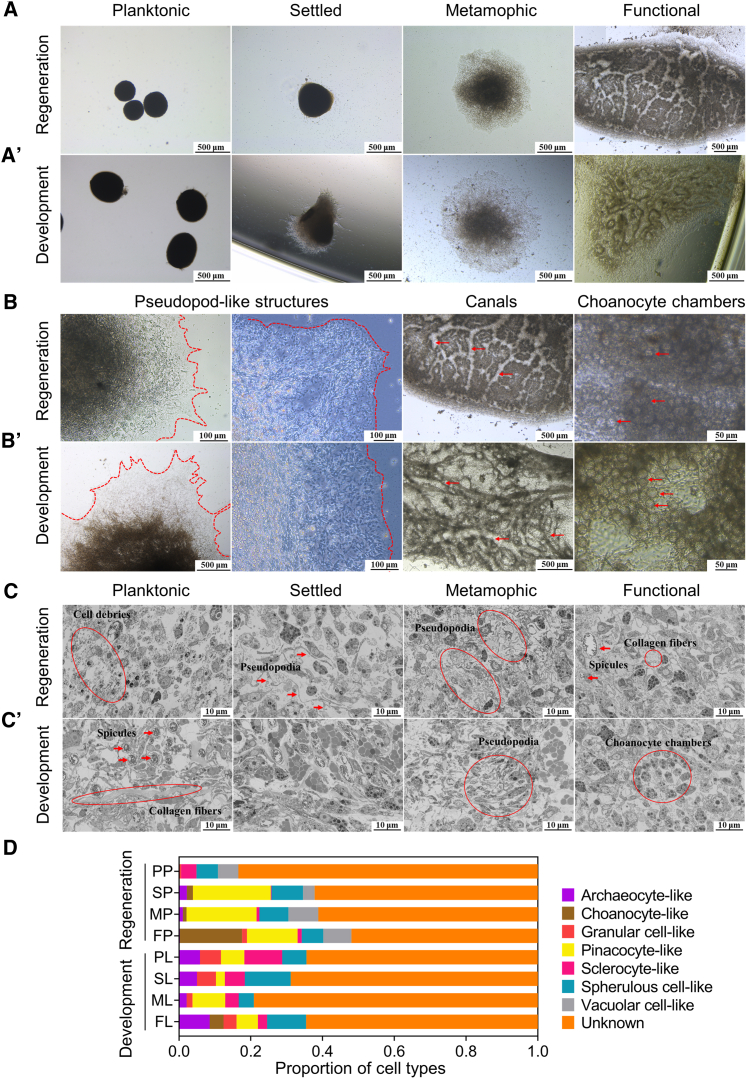
Morphological and cytological comparison of primmorph regeneration and larval development in *H. simulans* (A and A′) Morphological events of primmorph regeneration (A) and larval development (A′). Scale bars, 500 μm. (B and B′) Characteristic features including pseudopod-like structures (red dashed lines), canals (red arrows), and choanocyte chambers (red arrows) of primmorphs (B) and larvae (B′). Scale bars (50, 100, 500 μm) are indicated in the lower right corner of each micrograph. (C and C′) Ultrastructure of primmorphs (C) and larvae (C′). Cell debris, pseudopodia, spicules, collagen fibers and choanocyte chambers are indicated by red circles or arrows. Scale bars, 10 μm. (D) Proportion of cell types during regeneration and development, estimated from ≥500 cells across multiple fields per stage (*n* = 3 biological replicates). PP, SP, MP, and FP represent planktonic, settled, metamorphic primmorphs and functional individuals regenerated from primmorphs. PL, SL, ML, and FL represent planktonic, settled, metamorphic larvae and functional individuals developed from larvae. See also Figures S2, S3, and Table S1.

We established a scheme for whole-body regeneration of marine sponges which was applicable to demosponges including *Dysidea* sp., *H. simulans*, *M. phyllophila*, and *Niphates* sp. (Figure S1). Due to its dominant ecological niche in the southeastern coast of Fujian, *H. simulans* was selected for further study. Following adult tissue dissociation, the discrete cells could spontaneously aggregate into cell clusters (termed “primmorphs”) and then reconstruct fully functional individuals through a series of morphogenetic events (Figures 1A and 1B).

Planktonic primmorphs stage: Dissociated cells rapidly aggregated into primmorphs from 50 μm to 1 mm through cell incorporation and fusion (Figure 1A). These floating aggregates displayed smooth outer epidermis and compact structure, but internal ultrastructure exhibited loose packaging (Figure 1C). Dominant cell types included granular cells, sclerocytes, spherulous cells, and vacuolar cells, while archeocytes and choanocytes were not detected within the examined samples (Figures 1D and S2). Cells were observed to lose their original cellular morphology.

Settled primmorphs stage: planktonic primmorphs adhered to substrates without obvious morphological changes (Figure 1A). The previously morphologically ambiguous cells appeared to acquire an amoeboid morphology, as evidenced by more frequent pseudopodia formation, leading to tighter cellular arrangement (Figure 1C). Archeocytes, choanocytes, and pinacocytes reemerged alongside persistent granular cells, sclerocytes, and vacuolar cells (Figures 1D and S2).

Metamorphic primmorphs stage: attached primmorphs flattened significantly, accompanied by pinacoderm expansion (Figure 1B) and active pseudopodia facilitating fusion (Figure S3A). Internal reorganization featured compacted cellular arrangements (Figure 1C) and nascent lacunae forming aquiferous systems (Figure S3B). Cell populations remained stable (Figure 1D).

Functional individual stage: mature primmorphs reconstructed into functional juveniles, characterized by canal networks and functional choanocyte chambers (Figure 1B), with particulate flow through canals confirming the filter-feeding function of the aquiferous system (Video S1). Internal cell morphology and types resembled the metamorphic primmorph stage (Figure 1D).

Video S1. Filter-feeding in the aquiferous system of functional individuals reconstructed by primmorphs in *H. simulans*

Primmorph regeneration and larval development functionally converged to generate filter-feeding juveniles and produced morphologically analogous structures, including pseudopod-like extensions, choanocyte chambers, and aquiferous systems (Figures 1A, 1A′, 1B, and 1B′). Behaviorally, primmorphs also exhibited characteristics similar to those of larvae, such as inter-individual fusion during morphogenesis (Figures S3A and S3D) and reversible metamorphosis under environmental stress (Figures S3C and S3E). Cytologically, however, two processes exhibited divergence. Early primmorph regeneration involved fragmented, loosely packed cells that progressively compacted through pseudopod formation (Figure 1C). Conversely, larval development maintained consistent cell morphology and compact organization throughout all stages (Figure 1C’). Notably, archeocytes and choanocytes were temporarily rare or undetectable during the planktonic phase of primmorph regeneration but reemerged during settlement, accompanied by a marked increase of pinacocytes (Figure 1D). Vacuolar cells, which were found to be scarce in larval stages despite limited samples, persisted and even became a predominant cell population throughout regeneration (Figure 1D). The cellular composition and morphogenetic progression during the early phases of primmorph regeneration are thus distinct from those observed in larval development.

### Microbiome comparison between primmorph regeneration and larval development

**Figure 2:**
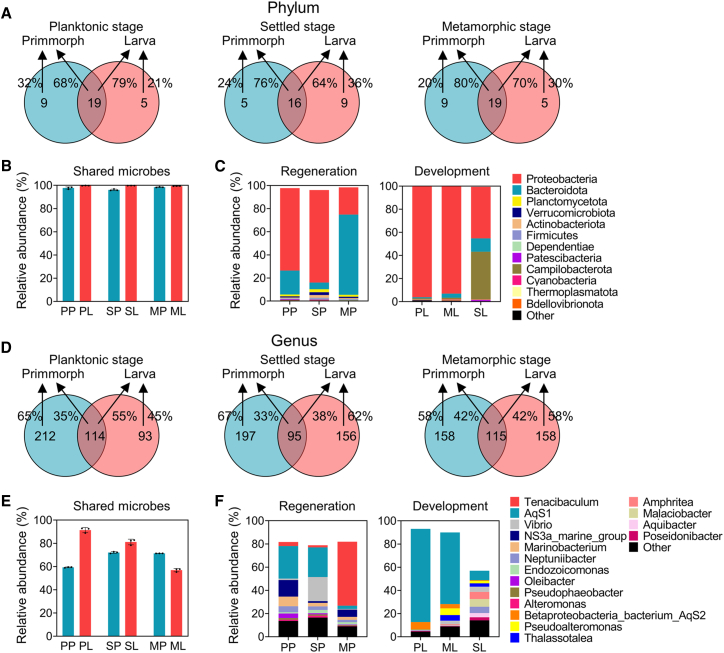
Microbial composition and their abundance trends during primmorph regeneration (*n* = 3 biological replicates) and larval development (*n* = 3 biological replicates) in *H. simulans* (A and D) Number and percentage of shared phyla (A) and genera (D) between primmorphs and larvae during planktonic, settled and metamorphic stages. (B and E) Relative abundance of shared phyla (B) and genera (E). Data are represented as mean ± standard deviation of the mean. (C and F) Relative abundance of phyla (C) and genera (F) during regeneration and development. Unclassified microorganisms are excluded. See also Tables S4 and S5.

Given the important role of microbial communities in sponge nutrient metabolism, immune defense, and developmental regulation, we further compared the microbial community characteristics during regeneration and larval development. The preprocessing and quality control of 16S sequencing data are presented in Tables S2 and S3. Comparative analysis of microbiomes during sponge primmorph regeneration (PP, SP, and MP) as well as larval development (PL, SL, and ML) revealed shared and process-specific microbial signatures. Primmorphs and larvae at the same stage harbored 16 to 19 phyla in common, accounting for 96.1% to 99.8% of the total microbial abundance (Figures 2A, 2B, and Table S4). The top 10 phyla in both primmorphs and larvae were largely consistent (Figure 2C). Proteobacteria dominated in early stages (PP: 71.3%, SP: 80.0%; PL: 96.0%, and SL: 92.9%) but declined significantly during metamorphic stage (SL: 23.6% and ML: 44.6%) (Figure 2C). At the genus level, 33%–55% of genera overlapped between primmorphs and larvae per stage, representing 56.9%–91.4% of community abundance (Figures 2D, 2E, and Table S5). Notably, only three genera (*AqS1*, *Vibrio*, and *Neptuniibacter*) ranked among the top 10 genera in both primmorphs and larvae (Figure 2F). Specifically, AqS1 exhibited a progressive decline in abundance during primmorph regeneration (28.1%, 25.5%, and 2.8%) and larval development (80.4%, 61.7%, and 8.1%), especially during the metamorphosis stage (Figure 2F).

**Figure 3:**
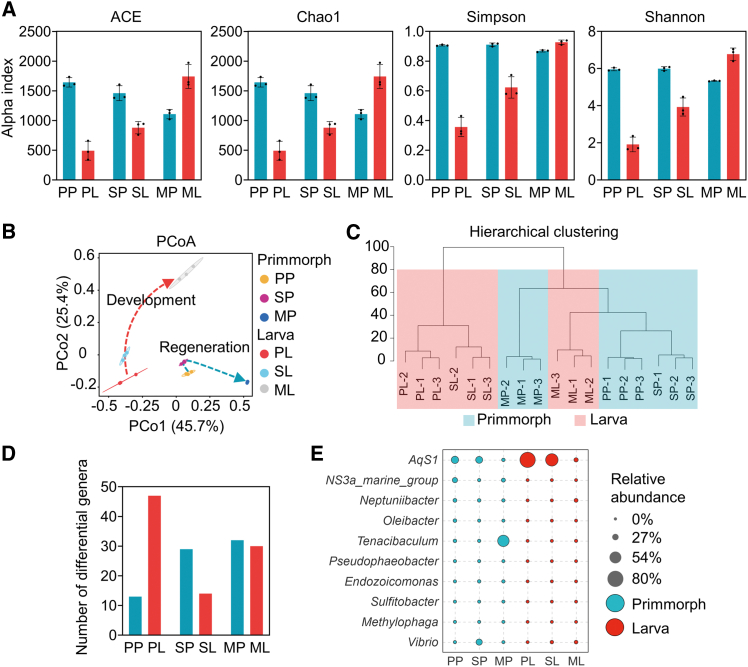
Comparison of microbial community structure between primmorph regeneration (*n* = 3 biological replicates) and larval development (*n* = 3 biological replicates) in *H. simulans* (A) Alpha diversity analysis. Microbial richness and diversity were assessed using the ACE, Chao1, Simpson, and Shannon indices. Data are represented as mean ± standard deviation of the mean. (B) Principal coordinate analysis based on Bray-Curtis dissimilarities. The dashed red and blue lines mark the microbial community progression during development and regeneration, respectively. (C) Hierarchical clustering analysis of microbial communities during primmorph regeneration (blue background) and larval development (red background) based on Bray-Curtis dissimilarities. (D) Number of differential genera between regeneration and development identified by Welch’s *t* test (*p* < 0.05). (E) Relative abundance of selected differential genera between regeneration and development identified by Welch’s *t* test (*p* < 0.05). The full list of differential genera is provided in Figure S4. See also Tables S6 and S7.

Despite a core set of shared microbes, the microbial community structure diverged between regeneration and development processes. Alpha diversity analysis showed contrasted trends: larval development exhibited increasing microbial richness and diversity, whereas primmorph regeneration showed declining richness with stable diversity (Figure 3A and Table S6). Despite the non-significant difference revealed by Adonis test (Table S7), principal coordinate analysis (PCoA) revealed a distinct primmorph-larva separation across stages (Figure 3B). Hierarchical clustering analysis indicated that this difference was minimized during metamorphic phase (Figure 3C). Despite the possible “convergence” in MP and ML stages, there still existed numerous differential microbes. Specifically, we identified 60 genera at the planktonic stage (*AqS1*, *AqS2*, *Marinobacterium*, etc.), 43 genera at the settled stage (*AqS1*, *AqS2*, *Vibrio*, etc.), and 62 genera at the metamorphic stage (*Tenacibaculum*, *Malaciobacter*, *NS3a_marine_group*, etc.) that were significantly different in abundance between the two processes (Figures 3D, 3E, and S4).

### Transcriptomics comparison between primmorph regeneration and larval development

**Figure 4:**
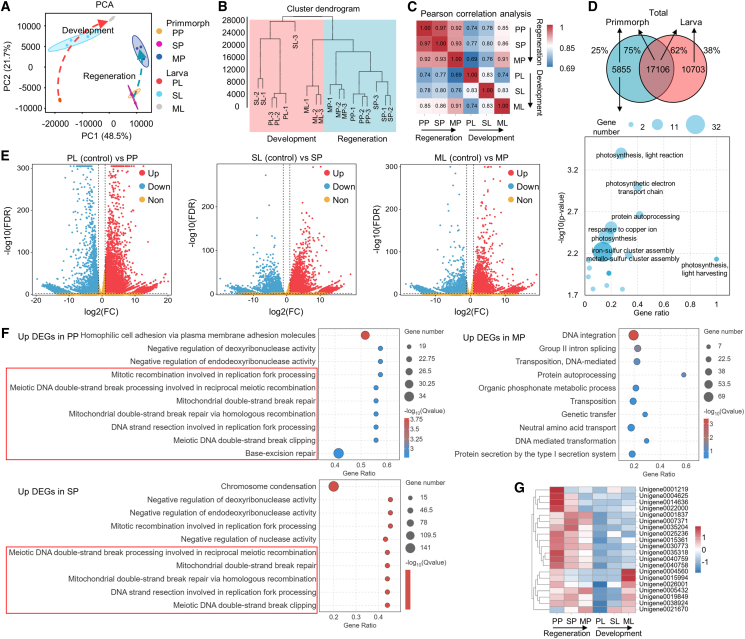
Comparison of regenerative (*n* = 3 biological replicates) and developmental (*n* = 3 biological replicates) transcriptomes in *H. simulans* (A) Principal component analysis. The dashed red and blue lines mark the overall expression trajectory of development and regeneration samples, respectively. (B) Hierarchical clustering analysis of transcriptomes from primmorph regeneration (blue background) and larval development (red background). (C) Heatmap of Pearson correlation coefficient between different samples. (D) Venn diagram (top) illustrating the number and proportion of expressed genes between primmorphs and larvae. Bubble plot (bottom) displaying the top 20 significantly enriched GO terms in the biological process category for primmorph-specific genes. Representative terms are annotated on the plot. (E) Volcano plot of DEGs between primmorphs and larvae (control) in different stages. Significantly upregulated (red) and downregulated (blue) genes are highlighted (an absolute value of log2 (fold change) ≥ 1; FDR <0.05). Non-significant genes are shown in orange. (F) GO analysis of the DEGs that are highly expressed in PP, SP, and MP compared to the larvae. GO terms related to DNA repair are marked with red boxes. (G) Heatmap of gene expression levels associated with DNA repair in top GO annotations of DEGs from PP vs. PL and SP vs. SL comparisons. See also Tables S11, S12, and S14.

Comparative transcriptome analysis of sponge primmorph regeneration (PP, SP, and MP) and larval development (PL, SL, and ML) revealed dynamic gene expression patterns. After removing low-quality data, each sample retained a total of 35,980,970 to 43,459,058 clean reads. Transcriptome sequencing quality and annotation are presented in Tables S8, S9, and S10. Principal-component analysis (PCA) and hierarchical clustering analysis indicated initial divergence between regeneration and development samples, with progressive convergence toward metamorphic stages (Figures 4A and 4B). Pearson correlation coefficients between primmorphs and larvae increased from planktonic (0.74) to settled (0.80) to metamorphic (0.91) stages (Figure 4C), underscoring transcriptomic harmonization during later stages.

The Venn diagrams demonstrated 17,106 genes shared between regeneration and development, representing 75% and 62% of their total expressed genes, respectively (Figure 4D). Gene ontology (GO) analysis of the 5,855 regeneration-specific genes revealed enrichment for terms such as photosynthesis, light reactions, and protein autoprocessing (Figure 4D). Among these, the top-expressed genes included *fibcd1-a*, *eprA1*, *orf1*, annotated as predicted ficolin-1-like isoform X2 involved in innate immunity, uncharacterized protein LOC105313503 isoform X1 in sponge *Amphimedon queenslandica*, and polyprotein-4 associated with viruses, respectively (Table S11).

The number of differentially expressed genes (DEGs) (an absolute value of log2 (fold change) ≥ 1 and false discovery rate (FDR) ≤ 0.05) gradually decreased from planktonic (14,833) to settled (10,019) to metamorphic (8,604) stages between regeneration and development (Figure 4E and Table S12), suggesting transcriptomic convergence at later stages. DEGs were enriched in top KEGG pathways, including the MAPK, Wnt, TGF, Notch signaling pathways and ECM-receptor interaction, all of which are known to be involved in cell differentiation and division (Figure S5 and Table S13). Within each pathway, gene expression remained relatively stable during primmorph regeneration but exhibited more fluctuations during larval development, especially with many genes highly expressed in planktonic stage (Figure S6).

GO enrichment analysis of DEGs highly expressed in primmorphs compared to larvae revealed that most of the top terms in the planktonic and settled stages are related to DNA repair and recombination, such as negative regulation of deoxyribonuclease activity, mitotic recombination involved in replication fork processing, and mitochondrial double-strand break repair, whereas those in the metamorphic stage are associated with material transport (Figure 4F and Table S14). Notably, these DNA repair-related genes were either consistently expressed at low levels throughout larval development or showed elevated expression only during metamorphosis (Figure 4G and Table S14). These findings suggested that DNA repair process could be particularly active during the initial stages of regeneration.

**Figure 5:**
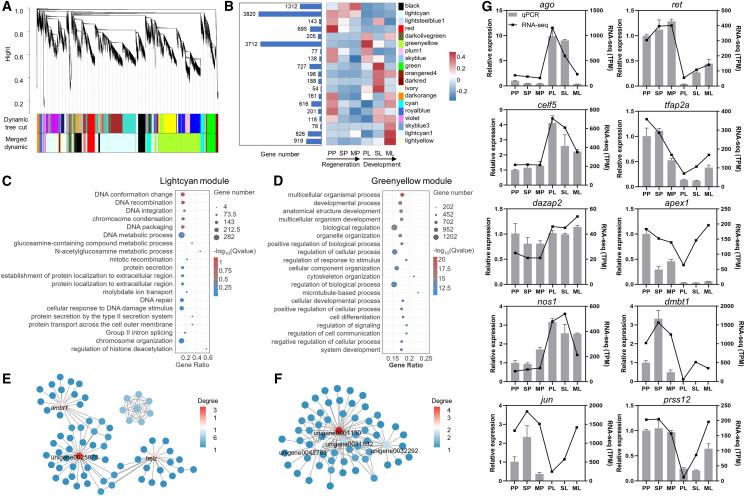
Analysis of co-expression modules and transcriptomic validation of DEGs (A) Hierarchical clustering dendrograms of identified co-expressed genes in modules. Each module is denoted by a distinct color, with individual genes represented as lines in hierarchical clustering. The height on the *y* axis indicates the distance between two genes. (B) The number of genes in each module (left) and the expression pattern of the module in each sample (right). The colors represent the normalized expression levels of the modules. (C and D) GO analysis of biological processes for genes in lightcyan (C) and greenyellow modules (D). (E and F) Networks of the genes with the top 100 connectivity rankings in gene-gene associations in lightcyan (E) and greenyellow modules (F). The color of the node indicates its degree. (G) Validation of ten DEGs associated with stem cell markers, DNA repair and other processes by RT-qPCR. The left y axis shows RT-qPCR relative expression calculated via 2^−ΔΔCT^ method, and the right *y* axis shows RNA-seq expression (TPM). Data are represented as mean ± standard deviation of the mean. *n* = 3 biological replicates. See also Figure S8, and Tables S15, S16, S17, and S18.

To further elucidate the molecular regulatory networks, we performed WGCNA on all genes with TPM >1 to identify co-expressed gene modules (Figures 5A and S7). A total of 14,188 genes were clustered into 19 correlated modules, each containing 54 to 3,820 genes (Figure 5B and Table S15). To identify the functional characteristics of key modules involved in regeneration and development, we conducted GO analysis on 8 modules with more than 500 genes (such as the lightcyan, greenyellow, and black modules) (Figures 5C, 5D, S8, and Table S15). The lightcyan module, which exhibited consistently high expression throughout regeneration, was enriched in DNA-related processes, including conformation change, recombination, integration, packaging, and metabolism, DNA repair, and cellular response to DNA damage (Figure 5C). The cyan module, showing high expression in both planktonic primmorphs and metamorphic larvae, was linked to cell proliferation and DNA damage repair (Figure S8). The red module, specifically upregulated in planktonic primmorphs, was associated with detection of mechanical stimulus (Figure S8). In contrast, modules specifically expressed during larval development, such as the greenyellow and green modules, were associated with the multicellular organismal and metabolic processes (Figures 5D and S8). Furthermore, we constructed a protein-protein interactions network of the expressed genes in lightcyan and greenyellow modules, and identified top hub genes based on degree (Figures 5E, 5F, and Table S16). The top hub genes in the lightcyan module were unigene0025878 (GO annotation indicated associations with chromosome segregation and condensation), *helz* (probable helicase with zinc finger domain), and *dmbt1* (deleted in malignant brain tumors 1 protein-like) (Figure 5E). The top hub genes in the greenyellow module were unigene0001130 (zinc transporter 2-like), unigene0042763 (ubiquitin carboxyl-terminal hydrolase 47-like), unigene0032292 (lacks valid annotation information), and unigene0031632 (uncharacterized protein) (Figure 5F).

The reliability of the RNA-seq data was validated by RT-qPCR analysis of ten key DEGs, which included stem cell markers (e.g., *ago*, *celf5*, *dazap2*, and *nos1*),^27^^,^^28^^,^^29^ DNA repair-associated genes (e.g., *apex1*, *dmbt1*, and *prss12*), and possible dedifferentiation-related genes (e.g., *jun*, *ret*, and *tfap2a*).^28^^,^^30^^,^^31^ (Figure 5G and Table S18). The relative expression patterns detected by RT-qPCR matched those from RNA-seq data. For example, the stem cell markers of marine invertebrates exhibited significant differences between the regeneration and development processes, with lower expression in primmorphs, and this trend was consistently recapitulated. Despite minor quantitative discrepancies, the overall strong concordance in relative expression patterns confirmed the accuracy of the transcriptomic data.

## Discussion

### Cellular plasticity underscores divergent pathways of sponge primmorph regeneration and larval development

Although sponge primmorph regeneration and larval development share external morphological and physiological similarities, they diverge markedly in internal structures and cell types, which is consistent with previous studies in Calcispongea.^7^ In contrast to larval development, planktonic primmorphs contain fewer archeocytes and choanocytes which are traditionally considered essential for pluripotency and differentiation in adult sponges.^27^^,^^28^^,^^32^ Nevertheless, these archeocyte and choanocyte-less primmorphs still regenerate into functional individuals, challenging the view that abundant archeocytes are prerequisites for sponge regeneration.^29^^,^^33^^,^^34^ Cells losing specific characteristics in planktonic primmorphs, followed by the subsequent appearance of archeocytes and choanocytes in settled primmorphs, implies possible dedifferentiation and transdifferentiation—conserved regeneration mechanisms in marine invertebrate regeneration.^20^^,^^28^^,^^30^^,^^31^ In contrast, larval development maintains relatively consistent cell morphology and proportion throughout all stages. Transcriptomic data further reflect this difference. Genes associated with germline multipotency program (*ago*, *celf5*, *dazap2*, and *nos2*) that are recognized as markers of sponge stem cells,^27^^,^^28^^,^^29^ are downregulated in primmorphs. While acknowledging the pleiotropic functions of these genes, primmorphs exhibit significantly elevated expression of genes such as *jun*, *ret*, and *tfap2a*, which have been implicated in promoting the reversion of differentiated cells to a progenitor-like state in other regenerate contexts.^35^^,^^36^^,^^37^ This expression pattern is not observed during larval development (Figure S9). Besides, vacuolar cells are consistently detectable throughout primmorph regeneration but are rarely observed during larval development. Although first detected here in this sponge primmorphs system, vacuolar cells are commonly associated with regenerative processes, including tissue damaged and budding.^38^ In sponge Homoscleromorpha species lacking archeocytes, vacuolar cells are predominant and exhibit high expression levels of germline pluripotency genes,^39^ implying they may possess archeocyte-like plasticity during regeneration. These findings highlight distinct pathways: primmorph regeneration involves dynamic reconstruction of cell populations, whereas larval development follows more conserved ontogenetic trajectories.

### Core microorganisms are implicated in sponge regeneration

Despite rigorous cell isolation protocols (mechanical dissociation, filtration, and centrifugation), core microbial communities are retained in sponge primmorph regeneration. Although primmorph regeneration and larval development share core microbiota, such as *AqS1*,^40^^,^^41^ their microbial succession diverges. *AqS1* is highly abundant in early primmorphs and larval stages but declines during metamorphosis, potentially regulating host development by compensating for host metabolic gene losses via arginine and purine provisioning.^42^ Furthermore, microbiome-transcriptome correlation analysis revealed that *AqS1* was strongly associated with the expression of 511 genes during primmorph regeneration (Figure S10 and Table S19). The top GO annotations were primarily related to photosynthesis-related processes, including photosynthetic electron transport chain, light reactions, and light harvesting (Figure S10), implying that *AqS1* may function in host energy metabolism. As the host’s nutrient reserves decline in early stage, this symbiont may be utilized as a resource during metamorphosis, possibly supporting host morphogenesis.

Microbial succession diverges between primmorphs and larvae, with variations in richness, diversity, and dominant taxa corresponding to the distinct contexts of regeneration and development. Primmorph regeneration is associated with an enrichment of *Vibrio* and *Tenacibaculum* species during the settled and metamorphic stages, which may degrade macromolecules and supply nutrients for host morphogenesis due to their metabolic versatility.^43^^,^^44^ This aligns with the documented roles of *T. mesophilum* in promoting larval settlement by various mechanisms,^45^^,^^46^^,^^47^ as well as that of *V. fischeri* in regulating development of the light organ in the squid *Euprymna scolopes*.^48^ Microbiome-transcriptome correlation analyses further suggest a potential association of *Tenacibaculum* with host energy metabolism (Figure S10). In contrast, larvae maintain relatively stable microbiomes (except for *AqS1*) via vertically transmitted symbionts and maternal nutrient reserves, thereby reducing reliance on exogenous microbial recruitment. Although sharing core taxa, the microbiome followed distinct restructuring trajectories: primmorph regeneration emphasized dynamic microbial turnover, while larval development conserved relatively stable symbionts. Nevertheless, despite the taxonomic differences throughout the process, the convergence of microbial community structure at metamorphosis suggests flexible host-microbe interactions that can achieve similar functional outcomes.

### DNA damage repair is activated during sponge early regeneration

Transcriptomics comparisons of *H. simulans* between primmorph regeneration and larval development reveal divergent early-stage gene expression patterns converging in the later stages, which are similar to calcareous sponges and sea anemones.^7^^,^^8^ In early primmorphs, DNA repair related GO terms were significantly enriched among upregulated DEGs. Some key hub genes in the lightcyan module, such as *helz* (a helicase involved in DNA double-strand break repair)^49^ and unigene0025878 (associated with chromosome segregation and condensation), maintained high expression levels throughout regeneration. This activation may be linked to the observed cellular fragmentation in early primmorphs, in contrast to the intact morphology of larvae. Animal regeneration involves rapid cell proliferation, genomic reprogramming, and tissue remodeling.^50^ These processes increase DNA replication and chromatin dynamics, potentially elevating the risk of DNA damage. Accordingly, the sustained expression of DNA repair genes possibly maintains genomic stability. Consistent with this, several genes encoding pleiotropic transcription factors (e.g., AP-2, LRRFIP, OTX, and ZF-C2H2) implicated in stem cell regulation^51^^,^^52^^,^^53^^,^^54^ were also upregulated in primmorphs, particularly during planktonic and settled stages (Figure S9). These findings align with studies in model organisms demonstrating that the lack of key DNA replication and repair components prevents regeneration in planarians,^55^ and homologous-mediated DNA repair is required for axolotl limb regeneration.^56^ Furthermore, microbiome-transcriptome correlation analysis indicated that the psychrophilic bacterium *Glaciecola* with minimal relative abundance during larval development (0%, 0%, and 0.01%) exhibited strong gene-level correlations with DEGs during primmorph regeneration (Figure S9). The top GO annotations of these genes were associated with DNA repair-related processes (e.g., meiotic DNA double-strand break processing, meiotic intra-S DNA damage checkpoint signaling). Given that microorganisms surviving in extreme environments generally possess enhanced DNA repair systems to withstand environmental-induced DNA damage, this correlation raises the possibility of a functional association between *Glaciecola* and host DNA repair activity during regeneration.^57^ These results highlight a key molecular difference between regeneration and development: the early and sustained activation of DNA repair pathways appears characteristic of regenerative processes, likely contributing to the maintenance of genomic integrity during extensive cellular restructuring.

### Insights into regeneration from sponge primmorph

There appears to be no “one size fits all” cellular mechanism involved in regeneration. Instead, various cellular strategies, including cell division, dedifferentiation, transdifferentiation, and stem cell activation, rely on the plasticity of multipotent or pluripotent cells.^58^ Under extreme conditions, sponges may replenish their stem cell reservoir for whole-body regeneration, potentially involving cellular reprogramming processes. Similar mechanisms are conserved in Echinodermata,^59^ Annelida,^31^ Chordata,^60^ and Amphibian,^61^ but are rarely observed in mammalian tissue repair, which relies more on the expansion and maturation of intrinsic stem cells.^62^ Although gene functions are often pleiotropic, the coordinated upregulation of genes related to DNA repair and cellular plasticity during sponge regeneration suggests a possible mechanism to balance the need for cell plasticity against the risks of genomic instability. This balance may pose a challenge in mammals, where dedifferentiation involves chromatin remodeling and DNA replication, increasing the risk of DNA damage. Sponges may mitigate this risk by activating DNA repair genes such as *helz*. In contrast, mammalian cells lack such sustained DNA repair activation; their DNA damage response networks (e.g., the p53 pathway) tend to prioritize cell-cycle arrest or apoptosis over repair.^63^ Nevertheless, mammal also retains latent plasticity which can be awakened by exogenous stimuli, for example, extracts from regenerating salamander limbs have been shown to induce proliferation and dedifferentiation in mammalian myotubes.^64^ Therefore, we propose that further investigation into how sponges temporally coordinate DNA repair with cellular plasticity during regeneration, thereby replenishing stem cell pools without compromising genomic stability, could illuminate strategies to safely unlock the regenerative potential in mammals.

Animal regeneration involves coordinated responses among diverse host cell types, as well as potentially interactions with associated microorganisms. The evidences of abundant microbes within extracellular matrix and even intracellular compartments of sponges, along with the fluctuation of specific microbial groups during whole-body regeneration, raise the possibility that taxa such as *AqS1*, *Tenacibaculum*, and *Glaciecola* may contribute to the regenerative process. This view is supported by findings in the flatworm *Convolutriloba longifissura*, where regeneration may depend on cross-species molecular integration via regulated host-algal transcriptional responses.^65^ Furthermore, antibiotic-induced regeneration delays reported in planarians, sea cucumbers, sponges further emphasize microbial roles in regeneration.^66^ Although mammals primarily depend on intrinsic gene networks for regeneration, such as those governing stem cell regulation and proliferation, their associated microorganisms, particularly the gut microbiota, may act as important modulators through metabolic and immune pathway.^67^^,^^68^^,^^69^ Thus, deciphering these microbe-associated regenerative mechanisms in sponges, a typical symbiotic holobiont model, could help elucidate the roles of host-microbe interactions in regeneration. Moreover, it may provide insights for the development of microbial-based therapy in tissue repair for mammals in future.

In conclusion, this study demonstrates that sponge whole-body regeneration achieves morphological outcomes comparable to larval development, yet through distinct cellular, microbial, and transcriptional pathways. Primmorph regeneration is characterized by early cytological remodeling, including reduced stem cells, the presence of numerous seemingly dedifferentiating cells and persist vacuolar cells, implying features possibly consistent with a reliance on cellular plasticity rather than a strict developmental program. Although microbial communities in both processes share a core symbiont (*AqS1*), primmorphs regeneration involves dynamic restructuring with stage-specific enrichment of taxa like *Vibrio* and *Tenacibaculum*. Transcriptomically, early regeneration is marked by the downregulation of stemness-related genes, a pronounced activation of DNA repair-related genes, and upregulation of pleiotropic genes often linked with dedifferentiation. In general, the comparative analysis emphasizes that sponge regeneration leverages shared biological mechanisms within a unique overall framework to produce functional individuals, thus highlighting the flexibility of cellular reprogramming, microbiome dynamics, and adaptive gene expression. Our integrated comparative framework provides a comprehensive dataset and testable hypotheses for subsequent investigations into metazoan regeneration.

### Limitations of the study

We acknowledge that this study presents several limitations. First, our findings are specific to primmorph reaggregation—a radical form of sponge whole-body regeneration from dissociated sponge cells; thus, direct comparisons with other regenerative processes (e.g., bilaterian epimorphosis) require caution. Second, cell type identification currently relies on morphological characteristics. Consequently, the reported proportions should be considered indicative and await definitive validation with cell-specific molecular markers. The putative cellular dedifferentiation, alongside the reduction in stem cells, suggests a potential compensatory mechanism that requires validation through future lineage-tracing studies. Third, although high-abundance stage-specific microorganisms (e.g., *AqS1*) were detected during primmorph regeneration, their functional roles remain to be validated through culturomics and metatranscriptomics. Furthermore, transcriptomics revealed regeneration-upregulated possible DNA repair and dedifferentiation-related genes, but their precise regulatory roles await experimental verification. Although this study is a preliminary investigation, it offers a comparative framework between primmorph regeneration and larval development, as well as foundational data for advancing sponge regenerative biology research.

## Resource availability

### Lead contact

Requests for further information and resources should be directed to and will be fulfilled by the lead contact Jing Zhao, sunnyzhaoj@xmu.edu.cn.

### Materials availability

This study did not generate any new reagents.

### Data and code availability

All data supporting findings of this study are provided within the manuscript and its supplemental information section. Raw sequencing data have been deposited at the NCBI Sequence Read Archive (SRA) under BioProject accession number: SRA: PRJNA1182027 (16S rRNA-seq data of primmorphs), SRA: PRJNA1191552 (16S rRNA-seq data of larvae), SRA: PRJNA1182074 (RNA-seq data of primmorphs), and SRA: PRJNA1182098 (RNA-seq data of larvae). This paper does not report original code. Any additional information required to reanalyze the data reported in this paper is available from the lead contact upon request.

## Acknowledgments

This research was supported by the 10.13039/501100003392Fujian Provincial Natural Science Foundation of China (2024J01018); the Fundamental Research Funds for the Central Universities (20720200045); and the 10.13039/501100001809National Natural Science Foundation of China (41876183). We would like to thank Professor Ming Chen for providing sponge experimental materials and Genedenovo Biotechnology Co., Ltd. (Guangzhou, China) for sequencing assistance.

## Author contributions

Conceptualization, C.J. and J.Z.; methodology, C.J., B.Z., and J.Z.; investigation, C.J., B.Z., Y.Z., and B.G.; writing—original draft, C.J.; writing—review and editing, J.Z.; funding acquisition, J.Z.; resources, X.L. and J.C., and J.Z.; supervision, C.J. and J.Z.

## Declaration of interests

The authors declare no competing interests.

## STAR★Methods

### Key resources table

**Table:** 

REAGENT or RESOURCE	SOURCE	IDENTIFIER
**Biological samples**		

Sponge *Dysidea* sp. tissue	This study	–
Sponge *Haliclona simulans* tissue	This study	–
Sponge *Haliclona simulans* larvae	This study	–
Sponge *Mycale phyllophila* tissue	This study	–
Sponge *Niphates* sp. tissue	This study	–

**Chemicals, peptides, and recombinant proteins**		

Sodium chloride	Solarbio	Cat# S8210
Potassium chloride	Solarbio	Cat# P9921
Sodium sulfate	Solarbio	Cat# S5640
Sodium bicarbonate	Solarbio	Cat# S5240
Tris-HCl	MedChemExpress	Cat# HY-D0227F
Ethylenediaminetetraacetic acid disodium salt	Solarbio	Cat# E8030
Glutaraldehyde	Solarbio	Cat# P1126
Ethanol	Sangon	Cat# A500737
Trichloromethane	Sangon	Cat# A375084

**Critical commercial assays**		

FastDNA™ SPIN Kit for Soil	MP	Cat# 116560200
Qiagen miRNeasy Mini Kit	Qiagen	Cat# 217004
PrimeScript RT reagent Kit with gDNA Eraser (Perfect Real Time)	Takara	Cat# RR047A
TB Green® Premix Ex Taq™ II	Takara	Cat# RR820A

**Deposited data**		

16S rRNA-Seq data from primmorphs deposited at SRA	This study	NCBI Sequence Read Archive: PRJNA1182027
16S rRNA-Seq data from larvae deposited at SRA	This study	NCBI Sequence Read Archive: PRJNA1191552
RNA-Seq data from primmorphs deposited at SRA	This study	NCBI Sequence Read Archive: PRJNA1182074
RNA-Seq data from larvae deposited at SRA	This study	NCBI Sequence Read Archive: PRJNA1182098

**Oligonucleotides**		

qPCR primer for the *sdha* gene	F: TAAACTGTTCCCTACTCGCTCAC R: TCTTCTGTCATGTAGTGGATGGC	–
qPCR primer for the *ago* gene	F: GGAATGCCGATACTGGCGAA R: CGAGTCAACGTCCCACACTC	–
qPCR primer for the *celf5* gene	F: CCGGCACCAAACAAGAAGAG R: TGCCTGTGCCTCCTGTCTA	–
qPCR primer for the *dazap2* gene	F: CCTGGCTATGCTCCCACAG R: ATACCCACCATCAGACCCTCC	–
qPCR primer for the *nos1* gene	F: CACTGGGGAAACATGACGCT R: CCATTGCTCCGGTCAGTTGT	–
qPCR primer for the *jun* gene	F: TGTACTCCTCTTATGCCCCTG R: TGCTTTCGGAGGTCGTTTGA	–
qPCR primer for the *ret* gene	F: AACTATATTGACCTCAGTGGCG R: CGGGTTGGTCTCTCGGTTT	–
qPCR primer for the *tfap2a* gene	F: AGACTGGGACTCAACAGACG R: ACGAGTGTCTCCCAGTGTGT	–
qPCR primer for the *apex1* gene	F: GTGCCATTGACCTTTCCTCTG R: ACACATCAGGGCACTCTTGA	–
qPCR primer for the *dmbt1* gene	F: GCAATGAAGGTGAGGTTCGTC R: TGCGGAGCGAGTAAAGTCA	–
qPCR primer for the *prss12* gene	F: TGCCTGCACTAGAAATGAGGAG R: TCACATAGACCATGCTACTGCC	–

**Software and algorithms**		

GraphPad Prism 8	Graphpad	https://www.graphpad.com/
OmicShare online tool	Genedenovo Biotechnology Co., Ltd. (Guangzhou, China)	https://www.omicshare.com/
Cytoscape 3.10.2	Cytoscape	https://cytoscape.org/
QuantStudio™ Real-Time PCR Software	Applied Biosystems	https://www.thermofisher.cn/

**Other**		

0.22 mm pore-size PVDF membrane filters	Millipore	ISEQ10100
Reef Crystals® Reef Salt	Instant Ocean	–

### Experimental model and study participant details

#### Adult sponges and larvae collection

To verify the availability of the protocol for whole-body regeneration of primmorphs *in vitro*, healthy specimens of *Dysidea* sp., *H. simulans*, *Mycale phyllophila*, and *Niphates* sp. were collected from a sponge mariculture area in Dongshan Bay, Fujian, southeast of China. All sponge specimens used in this study were cultured under relatively controlled conditions rather than being sourced from the wild, ensuring consistency in species throughout the study. In subsequent comparative analysis experiments, we chose *H. simulans* for both primmorphs and larvae preparation due to its dominant ecological niche in the southeastern coast of Fujian and its stability in primmorphs culture *in vitro*. The samples used in larvae and primmorphs experiments originated from the same individual sponge to minimize the effect of environmental and individual differences on the data. For larvae collection, our previous methods were used.^45^^,^^46^^,^^47^ We defined the different developmental stages of larvae according to the morphological characteristics and status observed by a biological inverted microscope, including free-swimming planktonic larvae (PL), settled larvae (SL) attaching to the bottom and remaining stationary, metamorphic larvae (ML) flattened with rapid extension of the pinacoderm, and functional individuals developed from larvae (FL) characterized by the aquiferous system. Larvae in different stages were sampled after being rinsed frequently with FSW to minimize contamination, then rapidly frozen in liquid nitrogen and stored at −80°C.

#### Primmorphs preparation and collection

After being washed frequently with FSW to minimize contamination, the sponge was cut into small pieces. The resulting tissue was mechanically squeezed through 300 nylon mesh (50 μm) into CMFSW-E. Following centrifugation at 800*g* for 10 min at 4°C, the pellet was resuspended in CMFSW. Repeat twice and then dissolve the precipitate with FSW. Afterward, the cell suspension was diluted to a concentration of 10^7^ cells/ml and cultured in Petri dishes at room temperature. In this study, we performed primmorph cultures from sponges *Dysidea* sp., *H. simulans*, *M. phyllophila* and *Niphates* sp. to validate the effectiveness of the protocol for whole-body regeneration in marine sponges. Based on microscopic observations, we defined different stages of regeneration according to their morphological characteristics. Specifically, multicellular aggregates with a smooth surface were identified as primmorphs. Suspended primmorphs were termed planktonic primmorphs (PP), those adhering to the bottom without obvious morphological changes were classified as settled primmorphs (SP), those further flattening to produce distinct pseudopod-like structures were identified as metamorphic primmorphs (MP), and those exhibiting the aquiferous system were defined as functional primmorphs (FP). Primmorphs samples at different stages were washed frequently with FSW to remove contamination, then quickly frozen in liquid nitrogen and stored at −80°C.

### Method details

#### Reagents preparation

FSW was obtained by filtration (0.22 μm) of natural seawater collected in Dongshan Bay, Zhangzhou City, Fujian Province, China. Calcium- and magnesium-free seawater (CMFSW) was prepared by dissolving 31.6 g NaCl, 0.746 g KCl, 0.994 g Na2SO4, 0.0168 g NaHCO3, 2.42 g Tris-HCl in 1 L distilled water, and CMFSW containing EDTA (CMFSW-E) was made with an additional 10 mM EDTA.^70^ Artificial seawater (ASW) was prepared by dissolving sea salt (Instant Ocean Reef Crystals) in distilled water and adjusting the salinity to 30 ppt.

#### Identification of ultrastructure and cell types inside primmorphs and larvae

To explore ultrastructure and cellular composition, samples representing eight critical stages of primmorphs (PP, SP, MP, and FP), and larvae (PL, SL, ML, and FL) were systematically collected, with approximately 30 specimens per stage. The specimens were fixed with 2.5% glutaraldehyde in ASW for 1 h, followed by three rinses in ASW. Subsequent to agar-embedding and ultrathin sectioning, the specimens were dehydrated in graded ethanol dilutions (30%, 50%, 70%, 80%, 95%, 100%). After critical point drying, the samples were coated with gold. Ultrastructural observations were conducted using a JSM-6390 LV SEM system, prioritizing backscattered electron detection to maximize cellular resolution and observe as many cells as possible. In this study, cellular identification followed the established sponge histology criteria, focusing on morphologically distinct cell types.^20^^,^^21^^,^^25^ Briefly, archeocytes-like were identified as large, spherical cells possessing a large nucleus with a prominent nucleolus; choanocytes-like exhibited an ovoid shape with a microvilli collar and a single flagellum, typically organized in choanocyte chambers; granular cells-like were recognized as spherical cells containing numerous small, electron-dense inclusions; pinacocytes-like displayed a characteristic T-shaped or spindle-like morphology; sclerocytes-like were identified by their irregular shape and close association with developing spicules; spherulous cells-like were defined as spherical cells containing large, electron-dense inclusions; vacuolar cells-like were distinguished by their irregular shape and the presence of one or more large, prominent vacuoles within the cytoplasm. The approximate percentage of various cell types in each stage was assessed by identifying at least 500 cells across multiple fields of view. It should be noted that morphological identification may have certain limitations, as some cell types exhibit overlapping morphological features, and subtle state differences may be overlooked without molecular validation.

#### DNA extraction, PCR amplification of 16S rRNA gene, and sequencing

Considering the convergence in forming functional individuals during later stages and the differences in early stages between primmorph regeneration and larval development, six stages of primmorph samples (PP, SP, and MP, *n* = 3 replicates per stage) and larval samples (PL, SL, ML, *n* = 3 replicates per stage) were further analyzed. The FastDNA SPIN Kit for Soil was used to extract DNA in triplicate according to the manufacturer’s instructions. DNA amplicon sequencing was carried out by Genedenovo Biotechnology Co., Ltd. (Guangzhou, China). Briefly, the primers 341F (CCTACGGGNGGCWGCAG) and 806R (GGACTACHVGGGTATCTAAT) were used to amplify the V3-V4 region of the 16S rRNA gene. After PCR product separation, purification, and quantification, the sequencing library was constructed, and Illumina Novaseq 6000 was used for sequencing.

#### Microbiome data analysis

Further bioinformatic processing followed the DADA2 Workflow. Briefly, raw data with adapters or low-quality reads were removed by FASTP.^71^ Paired-end reads were merged as raw tags using FLASH with a minimum overlap of 12 bp and a mismatch error rate of 2%.^72^ Further sequence filtering, dereplication, and denoising were conducted by DADA2 R package.^73^ The UCHIME algorithm was used to identify and delete chimerism.^74^ Finally, the amplicon sequence variants (ASVs) were acquired. Taxonomic assignment was performed using a Naive Bayes RDP classifier based on the Silva database.^75^ Alpha diversity indices (ACE, Chao1, Shannon, and Simpson) were calculated in QIIME.^76^ Beta diversity analysis was conducted using Principal Coordinate Analysis (PCoA), Adonis test (Permanova) and hierarchical clustering based on Bray–Curtis dissimilarities, and significant differences in relative abundance of differential microbes among groups were detected by Welch’s *t* test based on the criteria of *p* value <0.05, all of which were performed in the R project Vegan package.^77^ We also used the OmicShare online tool (https://www.omicshare.com/) for the above analysis and figure drawing.

#### RNA extraction, library construction, and sequencing

Total 18 samples (*n* = 3 replicates per stage) including six stages of primmorphs (PP, SP, and MP) and larvae (PL, SL, and ML) were selected for RNA sequencing. Total RNA was extracted using the micro RNA isolation kit (Qiagen). The quality and purity of RNA were determined by using Agilent 2100 (Agilent Technologies, USA). Enrichment and fragmentation of mRNA and reverse transcription to first-strand cDNA were conducted by oligo (dT), high temperature, and a reverse transcription enzyme mixture system, which distinguished the sponge host’s RNA from that of prokaryotic microorganisms. End repair and A-tailing were completed when synthesizing the second strand of cDNA. Then adapters were connected, and Hieff NGSDNA Selection Beads were employed for purification to select target fragments. Finally, the PCR library amplification was performed and sequenced using the Illumina Novaseq X Plus by Genedenovo Biotechnology Co., Ltd. (Guangzhou, China).

#### Transcriptome data analysis

To obtain high-quality clean reads, adapter-contaminated, low-quality (Q ≤ 20 for >50% of bases), and low-complexity reads (comprising >10% N bases or only A bases) were filtered using FASTP with default parameters.^71^ Transcriptome *de novo* assembly was carried out by Trinity.^78^ Assembly quality was assessed by the N50 value and Benchmarking Universal Single-Copy Orthologs (BUSCO) (http://busco.ezlab.org/). All unigenes were mapped to the NCBI Non-Redundant Protein Sequence Database (NR) (https://ftp.ncbi.nlm.nih.gov/blast/db/FASTA/), protein sequence annotation and review database (SWISS-PROT) (https://www.uniprot.org/), Clusters of Orthologous Groups of proteins (COG/KOG), and Kyoto Encyclopedia of Genes and Genomes (KEGG) using BLASTx (http://www.ncbi.nlm.nih.gov/BLAST/) with an E-value threshold of 0.00001. Transcription factor (TF) families were predicted by Animal TFdb (https://guolab.wchscu.cn/AnimalTFDB4/#/). Subsequently, functional annotations were derived according to the best alignment results.

We used the high-quality clean reads counts data as the initial input for DeSeq2 to identify differentially expressed genes (DEGs) based on the criteria of FDR < 0.05 and an absolute value of log_2_ (fold change) ≥ 1.^79^ Given our focus on genes involved in the regeneration process, we designated larval development as the control groups and primmorphs regeneration samples as the experimental group to compare their gene expression profiles. Gene expression levels were estimated by transcripts per kilobase of exon model per million mapped reads (TPM). Principal component analysis (PCA), hierarchical clustering, correlation analysis, Gene Ontology (GO) enrichment analysis and Kyoto Encyclopedia of Genes and Genomes (KEGG) pathway analysis were conducted using the OmicShare online tool with default parameters (https://www.omicshare.com/). For enrichment analysis, the background genes set consisted of all genes detected in both larval development and primmorph regeneration samples, and a hypergeometric test was used to identify significantly enriched GO terms and pathways among DEGs relative to the background. Co-expression networks were constructed using weighted gene co-expression network analysis (WGCNA) (v1.47) package in R.^80^ A total of 14,188 genes (with TPM >1) were identified from 18 transcriptome samples derived from primmorphs and larvae. Gene expression data were imported into WGCNA to construct co-expression modules using the blockwiseModules automatic network construction function with default parameters, except for the following settings: power = 8, TOMType = unsigned, mergeCutHeight = 0.2, minModuleSize = 50. The soft-thresholding power was set to 8 based on the scale-free topology fit criterion (Figure S7). Functional characterization of each module was performed through GO enrichment analysis. To identify hub genes within the modules, gene co-expression networks were constructed using Cytoscape, incorporating genes from the top 100 connectivity-ranked gene pairs. Hub genes were then determined based on gene degree, defined as the number of direct interactions each gene exhibits within the constructed networks. To explore potential interactions between microbial communities and gene expression during regeneration, Pearson correlation coefficients were calculated between differentially abundant microbial taxa and DEG expression levels. Genes were defined as strongly associated with a given microbial genus if they met the statistical thresholds: the *p* value <0.05 and an absolute Pearson correlation coefficient >0.9. Additionally, the OmicShare online platform (https://www.omicshare.com/) was utilized for conducting the aforementioned analyses and generating visualizations.

#### Data validation using quantitative real-time PCR (qRT‒PCR)

The expression patterns of key DEGs from the transcriptome were validated by qRT-PCR, which was performed on the same RNA samples used for RNA-seq (PP, SP, MP, PL, SL, ML). RNA was reverse-transcribed into cDNA using PrimeScript RT reagent Kit with gDNA Eraser (Takara) and used as templates for qRT‒PCR. Gene-specific primers (key resources table) were designed for DEGs including stem cell markers (*ago*, *celf5*, *dazap2*, *nos1*), DNA repair-associated genes (*apex1*, *dmbt1*, *prss12*), and others (*jun, ret*, *tfap2a*), with *sdha* as the internal reference.[x] Reactions were carried out in 20 μL volumes using TB Green Premix Ex Taq II (Takara) on a QuantStudio 6 Flex system under the following conditions: 95°C for 30 s, followed by 40 cycles of 95°C for 5 s, 61°C for 30 s, and 72°C for 20 s. Each sample was run in triplicate. Relative expression was calculated via the 2^−ΔΔCT^ method.

### Quantification and statistical analysis

The statistical details are indicated in both method details and figure legends. Differences in microbial community structure were detected via Adonis test (Permanova). Significant differences in relative abundance of differential microbes among groups were detected by Welch’s *t* test. DEGs were identified based on the criteria of FDR <0.05 and an absolute value of log2 (fold change) ≥ 1. The *p* value <0.05 was considered a significant difference. ∗ indicates 0.01 < *p* < 0.05; ∗∗ indicates 0.001 < *p* < 0.01; ∗∗∗ indicates 0.0001 < *p* < 0.001; ∗∗∗∗ indicates *p* < 0.0001. The error bars in the figures represent the standard deviation.

## Supplemental information

Document S1. Figures S1–S9, Tables S1–S4, S6, S7, S10, and S12

Table S5. Relative abundance of microbes at the genus level during primmorph regeneration and larval development, relevant to Figure 2

Table S8. Summary statistics of transcriptome sequences

Table S9. Detailed data on Benchmarking Universal Single-Copy Orthologs (BUSCO) analysis

Table S11. 34 genes specifically expressed in primmorphs with high level of expression (TPM >50), relevant to Figure 4

Table S13. Detailed data on KEGG enrichment analysis of DEGs in PP vs. PL, SP vs. SL, MP vs. ML

Table S14. Detailed data on GO enrichment analysis of DEGs that are highly expressed in PP, SP and MP compared to the larvae, relevant to Figure 4

Table S15. Detailed data on WGCNA gene modules and GO enrichment analysis of module genes, relevant to Figure 5

Table S16. Detailed data on gene co-expression network analysis of greenyellow and lightcyan gene modules and hub genes, relevant to Figure 5

Table S17. Detailed data on gene expression of stem cell markers, DNA repair-associated genes, cell dedifferentiation-associated genes, and genes encoding transcription factors implicated in stem cell maintenance, relevant to Figure 5

Table S18. Detailed data on qRT-PCR (CT values) for the 10 DEGs, relevant to Figure 5

Table S19. Detailed data on microbiome-transcriptome correlation analysis and GO enrichment analysis of DEGs correlated with *Glaciecola*, *AqS1*, and *Tenacibaculum*
