# Supplementary material for: Divergent dynamics in whole-body regeneration and larval development of sponge Haliclonasimulans: cytobiology, microbiome, and transcriptomics
Source: iScience. 2026 Mar 13;29(4):115344. doi: 10.1016/j.isci.2026.115344 (PMC13058987; doi:10.1016/j.isci.2026.115344)
Supplement: Document S1. Figures S1–S9, Tables S1–S4, S6, S7, S10, and S12 [file mmc1.pdf]

iScience, Volume *29*

Supplemental information

Divergent dynamics in whole-body regeneration and larval development of sponge *Haliclona simulans*: cytobiology, microbiome, and transcriptomics

Chenzheng Jia, Beibei Zhang, Bifu Gan, Yuqing Zhao, Xin Lin, Jun Chen, and Jing Zhao

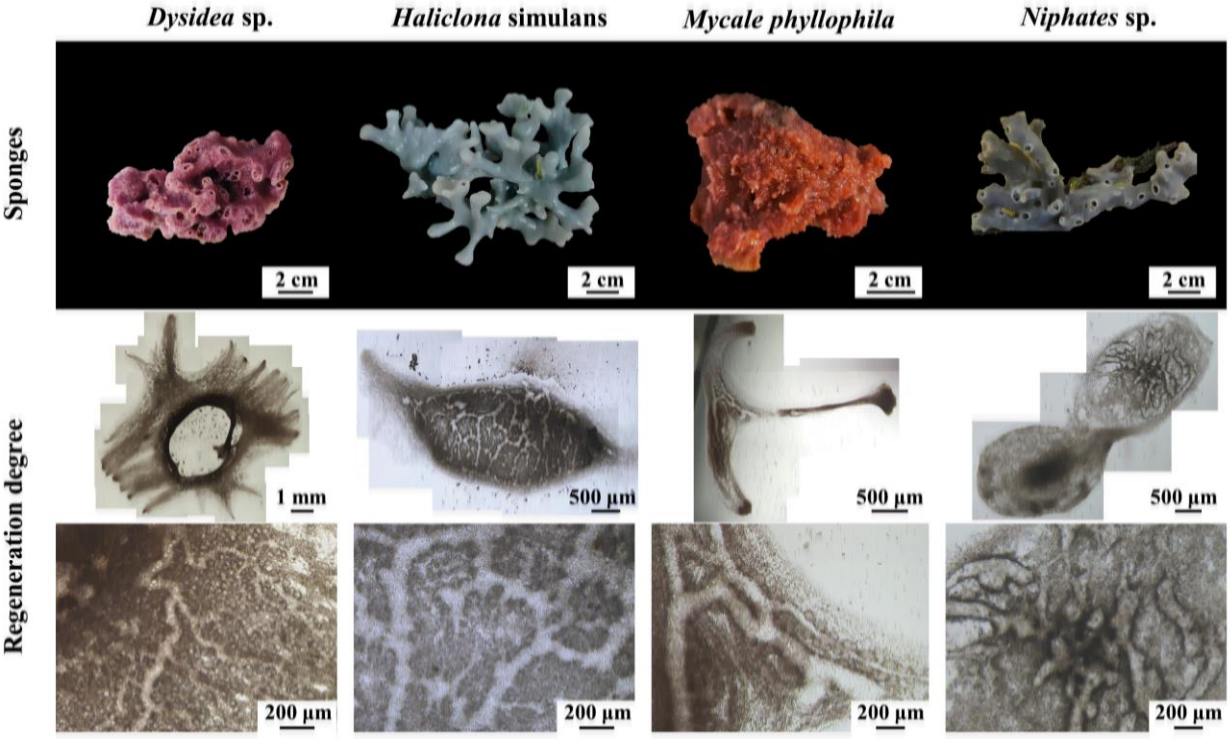

Figure S1. Whole-body regeneration of primmorphs in four sponge species including *Dysidea* sp., *H. simulans*, *M. phyllophila*, and Niphates sp.. The upper pictures show the morphology of four sponge species. The middle and lower pictures show that primmorphs generated from four sponge species reconstruct fully functional individuals characterized by aquiferous system. Scale bars are indicated at the bottom of each micrograph.

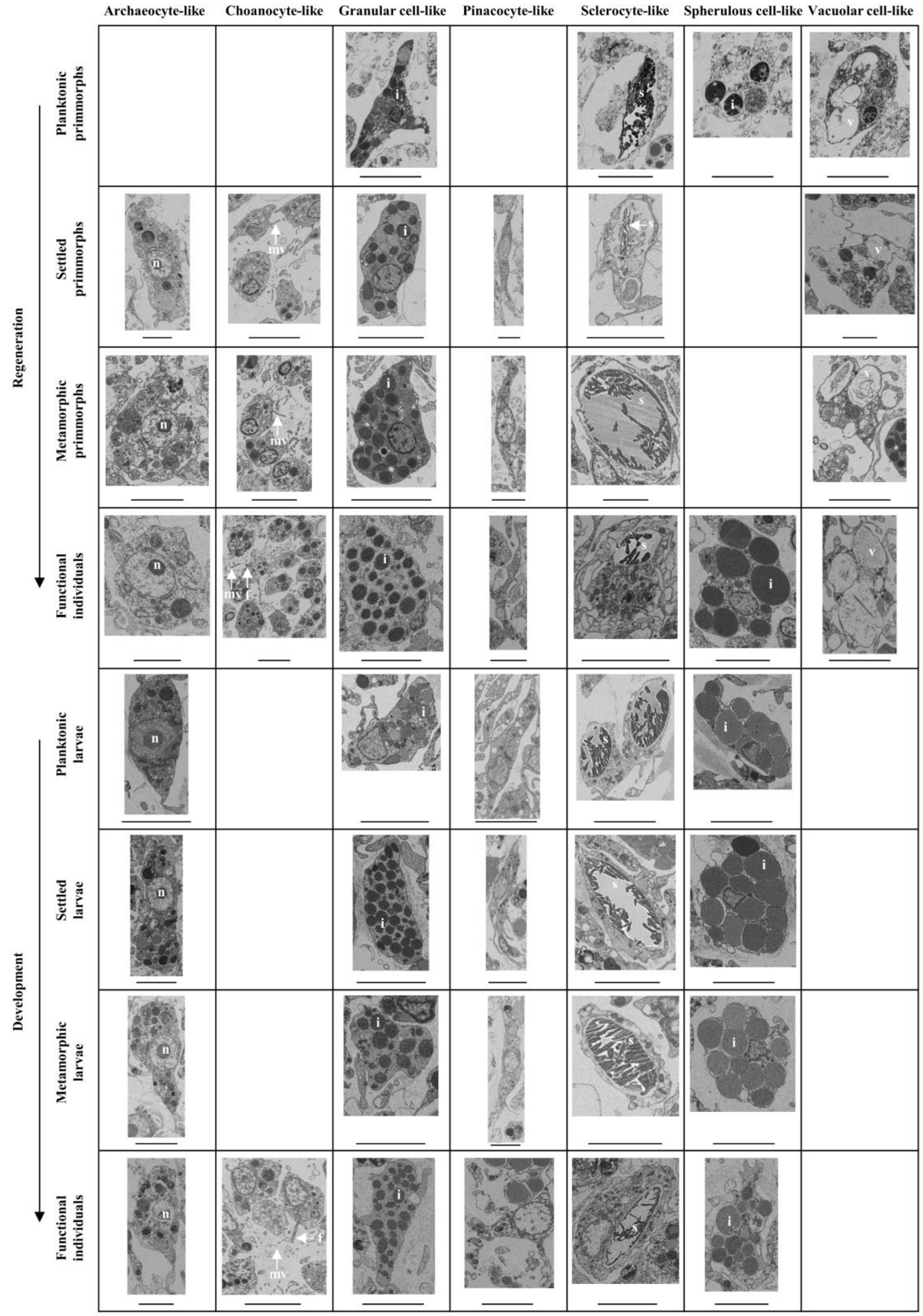

Figure S2. Cell types during primmorph regeneration and larval development in *H. simulans*. Various cell types, including archeocytes-like, choanocytes-like, granular cells-like, pinacocytes-like, sclerocytes-like, spherulous cells-like, and vacuolar cells-like were identified according to their typical characteristics. f, flagellum; i, inclusion; mv, microvilli; n, nucleus; s, spicule; v, vacuole. The scale bar is at the bottom of each cell morphology graph and represents 5 μm.

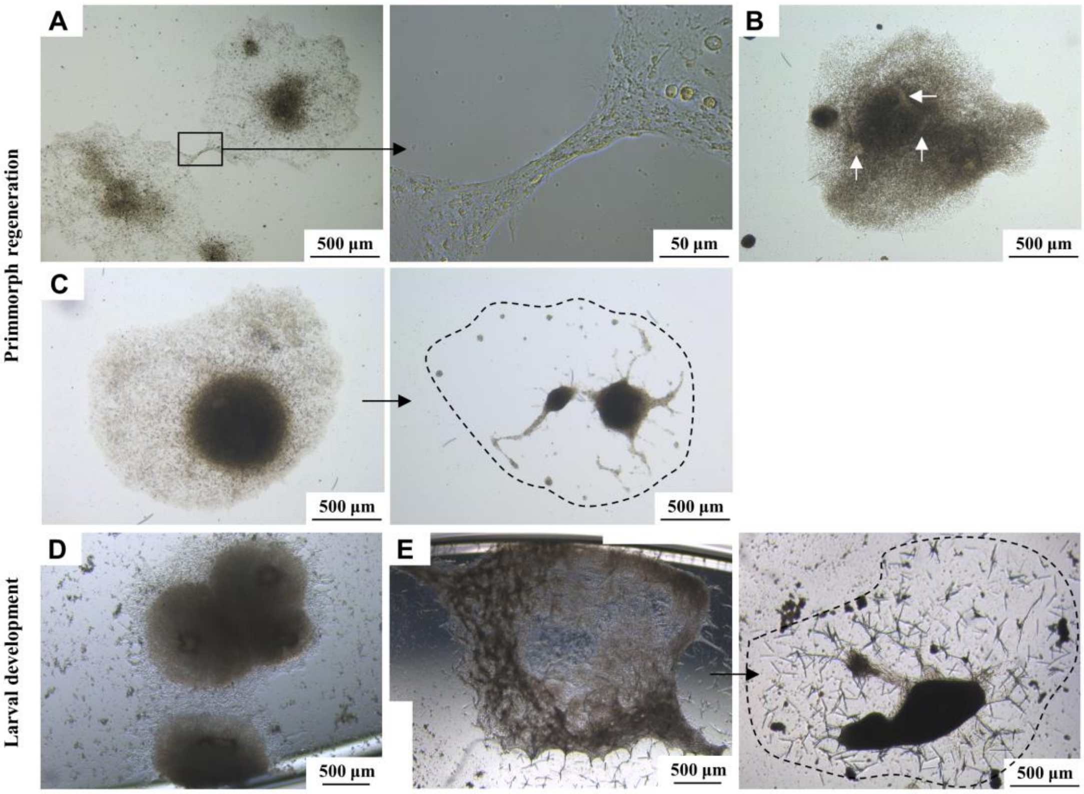

Figure S3. External morphology and physiological properties of primmorph regeneration and larval development in *H. simulans*.

(A) The formation of active pseudopod-like structures indicated by black box leads to the fusion of primmorphs. Enlarged picture is on the right.
(B) Small lacunae in primmorphs indicated by white arrows.
(C and E) Reversion from the metamorphic stage (left) to the settled stage (right) in primmorphs (C) and larva (E). The picture on the right shows the settled primmorph that is retracting the surrounding pseudopod-like structures into the center. The black dotted line indicates the area once covered by metamorphic primmorph.
(D) Fusion of settled larvae. Scale bars (50, 500 μm) are indicated in the lower right corner of each micrograph.

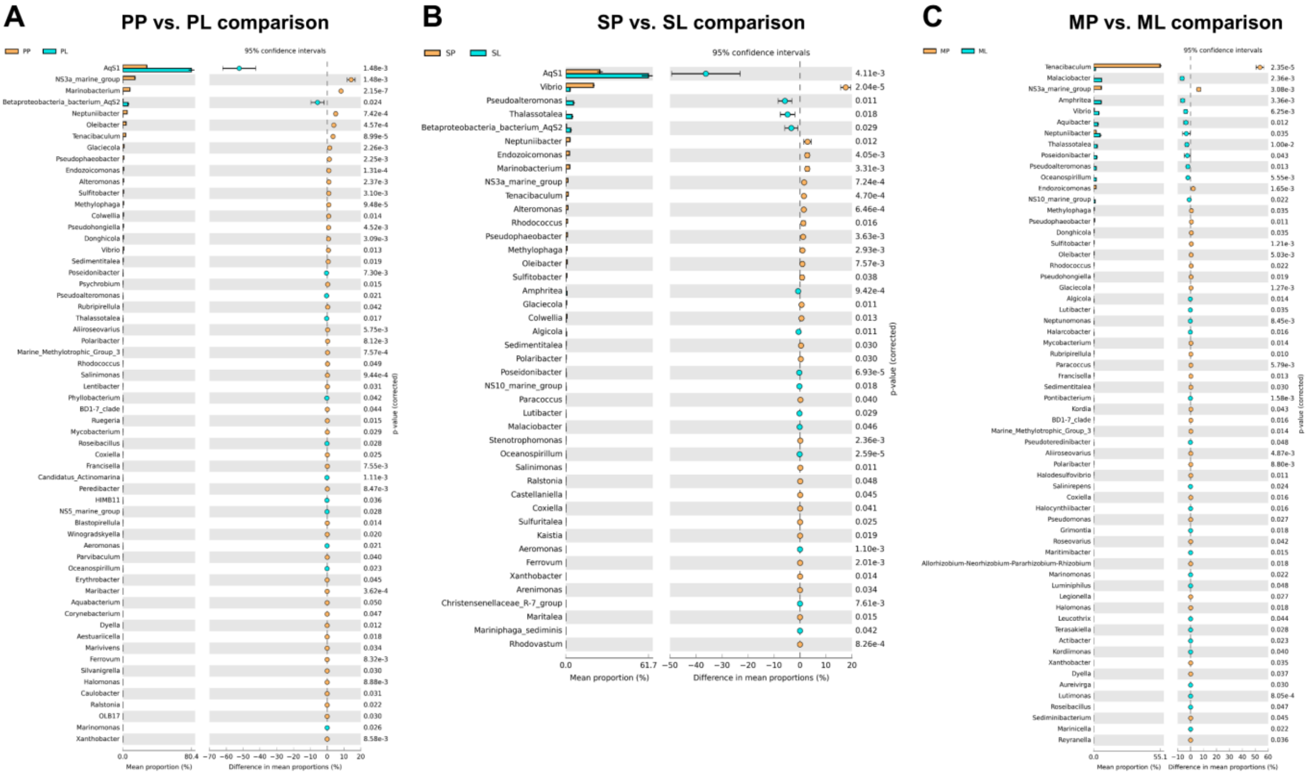

Figure S4. Analyses of microbes with relative abundance differences at the genus level.

(A, B and C) Analysis of differential genera between the primmorph regeneration and larval development, including PP vs. PL comparison (A), SP vs. SL comparison (B), and MP vs. ML comparison (C). Significant differences in relative abundance among different groups (n = 3 biological replicates) were analyzed by Welch's t-test ($p < 0.05$). The bar graph shows means ± standard deviation.

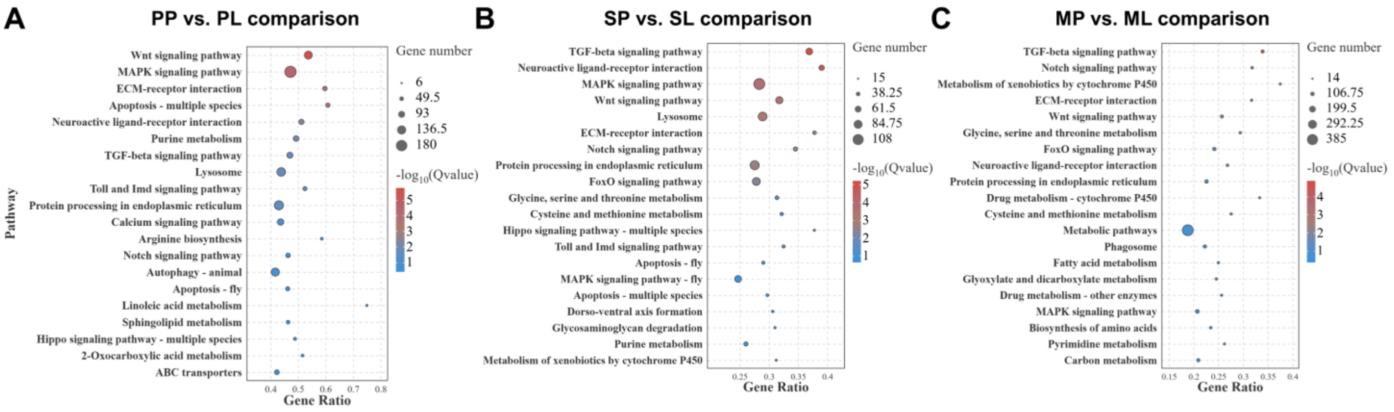

Figure S5. DEGs analyses between regeneration and development.

(A, B and C) KEGG enrichment analysis of DEGs in PP vs. PL (A), SP vs. SL (B), MP vs. ML comparison (C). n = 3 biological replicates.

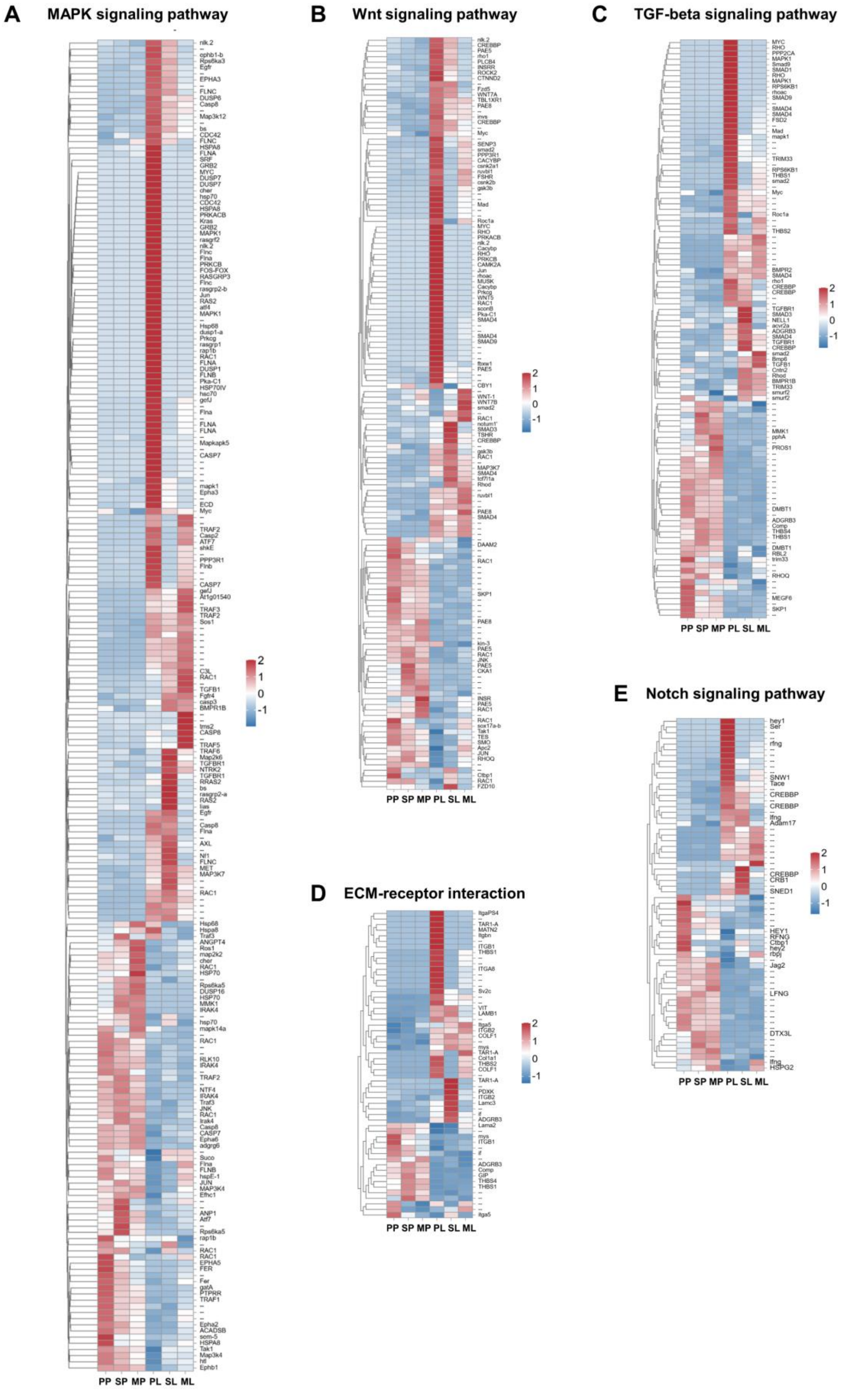

Figure S6. The expression patterns of DEGs in several key pathways including MAPK (A), Wnt (B), TGF-β (C), Notch (E) signaling pathways, and ECM-receptor interaction (D) (n = 3 biological replicates).

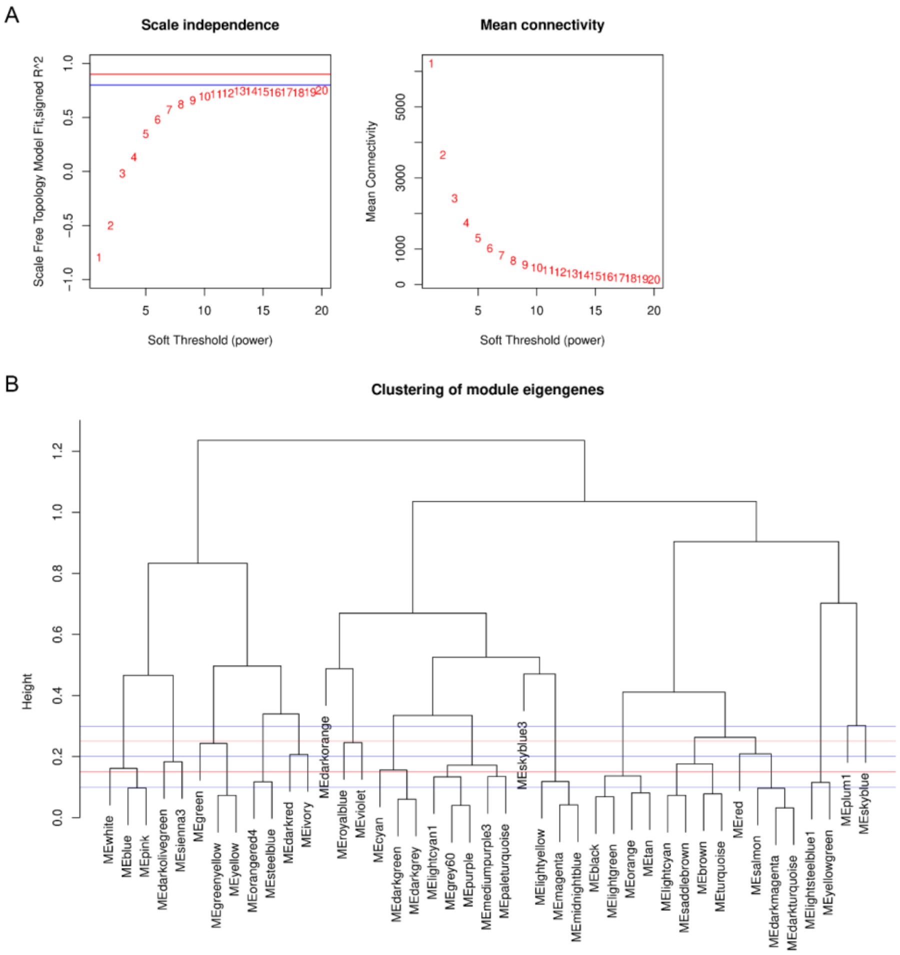

Figure S7. Network construction and module identification in WGCNA (n = 3 biological replicates).

(A) Analysis of network topology for various soft-thresholding powers. The scale-free fit index (left) and mean connectivity (right) were evaluated across different powers. Red and blue horizontal lines indicate the threshold lines for correlation values of 0.9 and 0.8, respectively.

(B) Clustering of module eigengenes. The inter-node distance reflects the correlation of module eigengenes. The height indicates the dissimilarity between modules, with lower values representing higher correlation. Colored lines beneath the dendrogram represent different height thresholds used to guide module merging, with modules below the threshold line being merged.

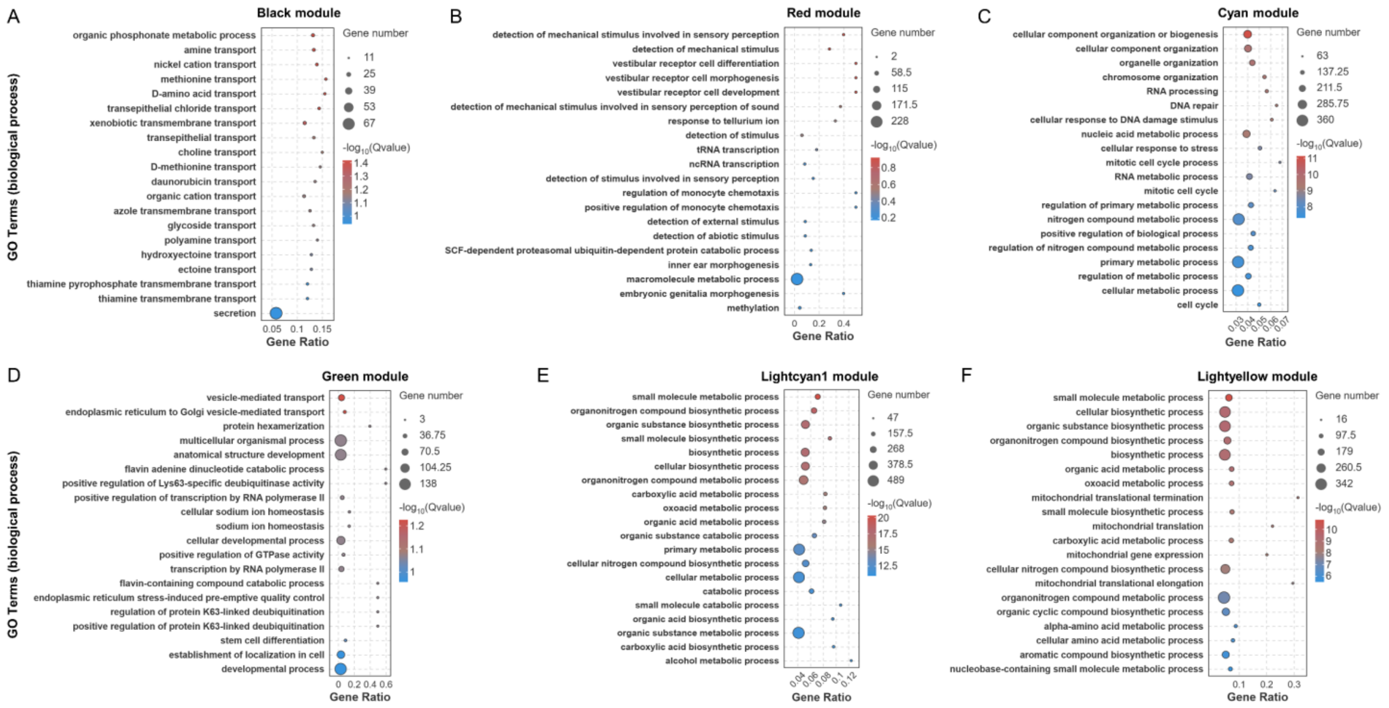

Figure S8. GO analysis of biological processes for genes in black (A), red (B), cyan (C), green (D), lightcyan1 (E) and lightyellow (F) modules. n = 3 biological replicates.

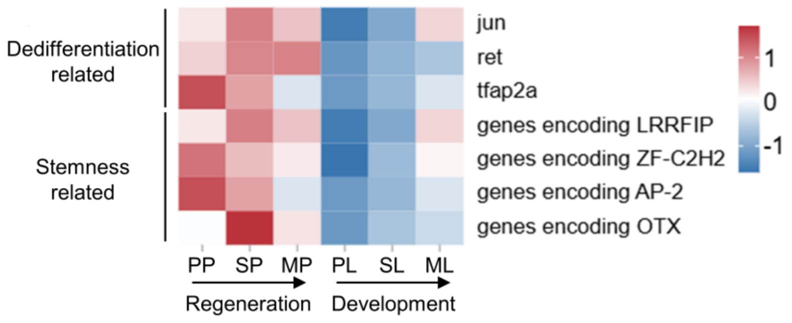

Figure S9. Heatmap of gene expression levels associated with dedifferentiation and stem cell maintenance-associated transcription factors. n = 3 biological replicates. See also Tables S17.

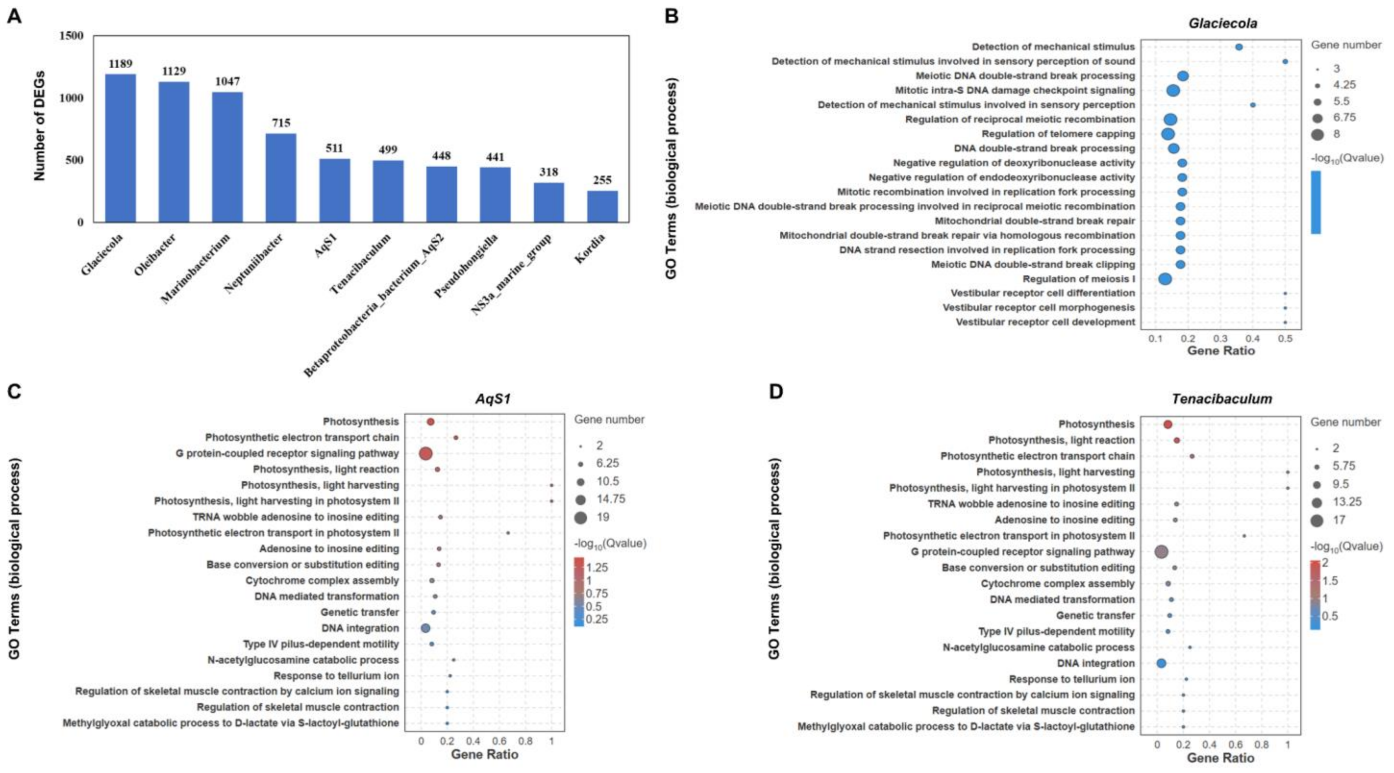

Figure S10. Microbiome-transcriptome correlation analysis (n = 3 biological replicates).

(A) Number of genes highly associated between the relative abundance of microbial genera and DEGs in the regeneration process.
(B, C and D) GO analysis of biological processes for Glaciecola-associated (B), AqS1-associated (C) and Tenacibaculum-associated genes (D).

Table S1. Quantitative data of proportion of cell types, relevant to Figure 1D.

	Archaeocyte-like	Choanocyte-like	Granular cell-like	Pinacocyte-like	Sclerocyte-like	Spherulous cell-like	Vacuolar cell-like	Unknown
PP	0.00	0.00	0.01	0.00	0.04	0.06	0.06	0.83
SP	0.02	0.02	0.00	0.22	0.00	0.09	0.03	0.62
MP	0.01	0.01	0.00	0.20	0.01	0.08	0.08	0.61
FP	0.00	0.17	0.01	0.14	0.01	0.06	0.08	0.52
PL	0.06	0.00	0.06	0.07	0.11	0.07	0.00	0.64
SL	0.05	0.00	0.05	0.03	0.06	0.13	0.00	0.69
ML	0.02	0.00	0.02	0.09	0.04	0.04	0.00	0.79
FL	0.09	0.04	0.04	0.06	0.03	0.11	0.00	0.65

Table S2. 16s sequencing data preprocessing and quality control.

Sample_ID	Raw_reads	Merged	Clean_reads	Denoised	Nonchimeras
PP-1	134386	133271	131237	124691	102892
PP-2	120843	119770	118107	111701	92503
PP-3	132145	130910	128800	121616	100251
SP-1	117535	116604	114711	107505	92941
SP-2	123555	122643	120809	114485	98186
SP-3	129058	128150	126125	119720	102918
MP-1	120605	119410	117494	112830	99665
MP-2	122809	121739	119763	114740	102351
MP-3	122936	121798	119933	115614	102905
PL-1	47505	47236	46750	45375	44800
PL-2	133543	132661	131277	128957	126346
PL-3	121724	120753	119373	117533	115849
SL-1	135102	134253	132938	130339	127388
SL-2	128661	127804	126688	123765	119429
SL-3	116931	116094	114883	111934	108409
ML-1	137068	135908	134733	125611	104982
ML-2	128388	127240	126224	118686	99467
ML-3	104849	104073	103223	97529	90223

Table S3. Detailed data on 16s sequencing tags.

Sample_ID	Tags number	Total length	Max length	Min length	N50
PP-1	102892	43496662	431	260	431
PP-2	92503	39191028	431	261	431
PP-3	100251	42504747	439	216	431
SP-1	92941	39399418	431	354	431
SP-2	98186	41460251	431	317	431
SP-3	102918	43439693	431	262	431
MP-1	99665	42129195	431	262	430
MP-2	102351	43246550	431	354	430
MP-3	102905	43463524	431	238	430
PL-1	44800	19174992	431	259	431
PL-2	126346	54101456	431	261	431
PL-3	115849	49639141	431	233	431
SL-1	127388	54497513	432	256	431
SL-2	119429	50942017	439	265	431
SL-3	108409	46379998	431	238	431
ML-1	104982	43799772	431	216	430
ML-2	99467	41468234	432	239	430
ML-3	90223	37582625	434	232	430

Table S4. Relative abundance of microbes at the phylum level during primmorph regeneration and larval development, relevant to Figure 2.

Phylum	PP	SP	MP	PL	SL	ML
Proteobacteria	71.32	80.04	23.56	95.98	92.85	44.64
Bacteroidota	20.70	5.79	69.40	0.97	3.59	11.53
Planctomycetota	1.54	2.56	1.68	0.12	0.32	0.07
Verrucomicrobiota	1.07	2.49	1.18	0.25	0.19	0.05
Actinobacteriota	0.69	2.30	0.91	0.28	0.15	0.12
Firmicutes	0.28	0.88	0.62	0.70	0.19	0.11
Dependentiae	0.40	0.53	0.40	0.00	0.00	0.00
Patescibacteria	0.70	0.38	0.06	0.03	0.14	1.15
Campilobacterota	0.44	0.40	0.19	0.94	1.35	41.28
Cyanobacteria	0.20	0.38	0.21	0.15	0.34	0.20
Bdellovibrionota	0.18	0.09	0.01	0.09	0.26	0.18
Desulfobacterota	0.04	0.08	0.16	0.07	0.04	0.03
Fusobacteriota	0.03	0.00	0.00	0.03	0.16	0.00
Others	0.13	0.17	0.03	0.14	0.08	0.03
Unclassified	2.26	3.91	1.58	0.25	0.34	0.61

Table S6. Detailed data on alpha diversity indies, relevant to Figure 3.

Index	ACE	Chao1	Shannon	Simpson
PP-1	1587	1587	5.90	0.90
PP-2	1612	1612	6.05	0.91
PP-3	1735	1735	5.94	0.91
SP-1	1607	1607	5.88	0.90
SP-2	1383	1383	6.03	0.92
SP-3	1396	1396	6.08	0.92
MP-1	1127	1127	5.32	0.87
MP-2	1175	1175	5.36	0.86
MP-3	1022	1022	5.34	0.87
PL-1	342	342	1.71	0.31
PL-2	660	660	2.37	0.43
PL-3	474	474	1.66	0.33
SL-1	761	761	3.56	0.58
SL-2	936	936	4.48	0.71
SL-3	944	944	3.74	0.58
ML-1	1973	1973	7.04	0.93
ML-2	1647	1647	6.40	0.91
ML-3	1606	1606	6.89	0.94

Table S7. Detailed data on statistical tests (Adonis test) for beta diversity, relevant to Figure 3.

Group	Df	SumOfSqs	R^2	F	Pr(>F)	P_adj_BH
PP-VS-SP	1	0.16	0.93	55.69	0.1	0.1
PP-VS-MP	1	0.47	0.99	332.49	0.1	0.1
PP-vs-PL	1	0.59	0.99	280.49	0.1	0.1
PP-vs-SL	1	0.48	0.96	99.91	0.1	0.1
PP-vs-ML	1	0.55	0.97	143.59	0.1	0.1
SP-VS-MP	1	0.55	0.98	178.06	0.1	0.1
SP-vs-PL	1	0.63	0.98	165.72	0.1	0.1
SP-vs-SL	1	0.49	0.95	75.09	0.1	0.1
SP-vs-ML	1	0.48	0.96	86.96	0.1	0.1
MP-vs-PL	1	1.17	0.99	504.59	0.1	0.1
MP-vs-SL	1	1.06	0.98	207.44	0.1	0.1
MP-vs-ML	1	0.75	0.98	183.93	0.1	0.1
PL-vs-SL	1	0.08	0.78	14.12	0.1	0.1
PL-vs-ML	1	0.99	0.98	207.19	0.1	0.1
SL-vs-ML	1	0.68	0.96	89.86	0.1	0.1

Table S10. Summary of annotation results for gene expression.

Total unigenes	NR	SWISSPORT	KOG	KEGG	Annotated unigenes	Unannotated unigenes
45594	22831	12422	22036	6624	33280	12314

Table S12. Number of DEGs during primmorphs regeneration (PP vs. SP and SP vs. MP comparisons), during larval development (PL vs. SL and SL vs. ML comparisons), and between regeneration and development (PP vs. PL, SP vs. SL, and MP vs. ML comparisons), relevant to Figure 4.

Comparisons	Up	Down	Total
PL vs. PP	8057	6776	14833
SL vs. SP	6155	3864	10019
ML vs. MP	4290	3774	8064
